# The communication of artificial intelligence and deep learning in computer tomography image recognition of epidemic pulmonary infectious diseases

**DOI:** 10.1371/journal.pone.0297578

**Published:** 2024-02-06

**Authors:** Weiwei Wang, Xinjie Zhao, Yanshu Jia, Jiali Xu

**Affiliations:** 1 Hangzhou Xinken Culture Media Co., Ltd., Hangzhou, China; 2 College of Media and International Culture, Zhejiang University, Hangzhou, China; 3 School of Software & Microelectronics, Peking University, Beijing, China; 4 Faculty of Science and Technology, Quest International University Perak, Ipoh, Perak, Malaysia; 5 School of Mathematics, Shanghai University of Finance and Economics, Shanghai, China; University of Oklahoma, UNITED STATES

## Abstract

The objectives are to improve the diagnostic efficiency and accuracy of epidemic pulmonary infectious diseases and to study the application of artificial intelligence (AI) in pulmonary infectious disease diagnosis and public health management. The computer tomography (CT) images of 200 patients with pulmonary infectious disease are collected and input into the AI-assisted diagnosis software based on the deep learning (DL) model, "UAI, pulmonary infectious disease intelligent auxiliary analysis system", for lesion detection. By analyzing the principles of convolutional neural networks (CNN) in deep learning (DL), the study selects the AlexNet model for the recognition and classification of pulmonary infection CT images. The software automatically detects the pneumonia lesions, marks them in batches, and calculates the lesion volume. The result shows that the CT manifestations of the patients are mainly involved in multiple lobes and density, the most common shadow is the ground-glass opacity. The detection rate of the manual method is 95.30%, the misdetection rate is 0.20% and missed diagnosis rate is 4.50%; the detection rate of the DL-based AI-assisted lesion method is 99.76%, the misdetection rate is 0.08%, and the missed diagnosis rate is 0.08%. Therefore, the proposed model can effectively identify pulmonary infectious disease lesions and provide relevant data information to objectively diagnose pulmonary infectious disease and manage public health.

## 1. Introduction

Epidemic pulmonary infectious disease is mainly transmitted through respiratory droplets and contact [1–3]. People are generally susceptible accompanied by symptoms, such as fever, cough, fatigue, or myalgia, and half of the patients can have dyspnea [4, 5]. Most cases are moderate or mild, the prognosis is good, and a few patients are critically ill and even die [6]. Apparently, epidemic pulmonary infectious diseases threaten human health. With the development of the epidemic, understanding of the epidemic pulmonary infectious diseases is deepening, especially, in terms of its epidemiology [7, 8]. Through effective and timely diagnosis under scientific public health management, more lives can be saved, patients’ symptoms will be alleviated, and the impact of the pandemic is minimized.

Computer tomography (CT) scan is an important diagnostic method and key to epidemic pulmonary infectious diseases patients with its high accuracy, convenience, good repeatability, and high positive rate, as well as its role in screening, disease progression evaluation, and public health management [9, 10]. Typical CT imaging features of pulmonary infection are multiple ground-glass opacity (GGO) with or without pulmonary consolidation on the periphery of both lungs, and early imaging examination is dominated by thin-layer high-resolution CT scanning. In such cases as pulmonary infectious disease, the patient’s condition changes constantly, resulting in multiple re-examinations of CT and massive amounts of images that increase the workload of physicians substantially. Due to the rich types of medical images, the lack of medical image data in specific fields is more prominent, restricting the effective development of research and practice. In this regard, Alzubaidi et al. (2020) put forward transfer learning and fine-tuning model training [11]. Setio et al. (2016) proposed a lung nodule detection method based on a convolutional neural network (CNN) algorithm. 76.00% sensitivity and 0.25 false-positive rate of CT per case were obtained in the test of the data set [12]. Different from traditional machine learning (ML) methods which need to extract artificial design features, deep learning (DL) can directly learn abstract and deep image features from raw image data [13]. Therefore, DL techniques have been used in large-scale computer-aided detection systems for chest CT pulmonary nodules.

With the rapid growth of computing power, memory storage, unprecedented computer rich data, and the development of advanced algorithms, artificial intelligence (AI) has exerted a significant role in dealing with new influenza, middle east respiratory syndrome coronavirus (MERS), and other epidemics. Rahman et al. (2022) [14] explored the role of emerging technologies such as AI and the Internet of Things (IoT) in Bangladesh’s public health response to infectious diseases. They analyzed the application of these technologies in the surveillance and control of infectious diseases and suggested related challenges and opportunities. Nousi et al. (2022) [15] demonstrated the application of data mining (DM) in dealing with epidemics through case studies. They described real-world AI-based applications, including predicting disease, monitoring vaccinations, and developing policies. They highlighted the potential and challenges of DM in pandemic response. Currently, AI can be applied to computer vision, speech recognition, natural language understanding (NLU), digital pathological data analysis, etc. In recent years, AI algorithms have been developed rapidly with the application of big data and the improvement of computer performance. Compared with the naked eye, AI technology can extract fine information details from the image, thus improving the diagnostic efficiency through the image. The application of AI technology to pulmonary nodule detection is the focus of the AI medical industry. To sum up, AI’s active response against pulmonary infectious disease is a revolutionary and innovative measure.

To improve the diagnostic efficiency and accuracy of pulmonary infectious disease, combined with DL target detection and image classification, this paper studies the CT signs of pulmonary infectious disease patients’ lesions and extracts and analyzes the features of lesion areas in different stages. The application value of the DL method in pulmonary infectious disease lesion screening and disease evaluation is analyzed. In general, the CT auxiliary diagnosis system based on DL and AI can well diagnose pulmonary infectious disease patients. Moreover, it can effectively identify lesions, provide relevant data information such as total lesion volume, internal GGO and solid change volume, and show changes in lesion extent and internal density.

## 2. Materials and methods

### 2.1. Research object

Two hundred patients: 106 males and 94 females, diagnosed with pulmonary infectious diseases in Hubei Hospital (From January 2020 to April 2020) were collected. The experiment was reviewed and approved by the Hubei hospital ethics committee, and all patients signed relevant informed consent. 200 pulmonary infectious disease images were saved in the final data set that was divided into the training set and test set: 80% of the images are used for training and the remaining 20% for testing. In addition to adjusting the size, enhancement operations should be performed on the training image to prevent network overfitting and help to remember the details of the training image. In the training set, the network is prevented from overfitting by resizing the image size to fit the model inputs and performing image enhancement operations, and helping the model remember the details of the training images. Preprocessing operations include grayscale, equalization, and filtering to enhance the features of the image. At the same time, when dividing the training and test sets, these 200 images of lung infectious diseases are saved in the final data set, and the ratio of male and female patients is kept similar to avoid the problem of category imbalance. A stratified sampling approach ensures that a sufficient number of samples for each class participate in training and testing. Such data set partitioning can help evaluate the model’s performance and make model selection. The training set ensures that the model can both learn the features of the data and make accurate predictions through the test set. DL models, specifically CNNs based on AlexNet models, are used in the training stage. Data from the training set are employed to tune the parameters of the model so that it can accurately identify pulmonary infection lesions. During the test stage, the performance of the model is evaluated. The images in the test set are 40 images of pulmonary infections in the dataset. Through reasonable dataset division and preprocessing operations, the diagnostic accuracy and generalization ability of the model for lung infectious diseases can be improved, and the research and application in the medical field can be further promoted.

Inclusion criteria: All patients met the diagnostic criteria of the national health commission’s *Pneumonia Diagnosis and Treatment Program for Novel Coronavirus Infection*, who showed Clinical manifestations of fever, cough, and fatigue; all patients completed high-resolution CT plain scan of the chest, and their clinic data were perfect.

Exclusion criteria: Patients with poor chest CT image quality, impact assessment, or no second CT examination, patients with negative nucleic acid detection, patients with lung cancer, severe interstitial pneumonia, pulmonary edema, or other causes of a severe pulmonary parenchymal lesion, and patients with incomplete clinical data.

### 2.2. CT scanning method

The CT examination of all patients was performed using a 64-row multi-layer spiral CT scanner (General Electric Company, USA). Two operation technicians were set up and carried out level 2 and above protection, one of them was responsible for patient placement, and the other one was responsible for scanning operation. Patients were allocated with exclusive compartments as much as possible. Each patient underwent a one-time single examination and was isolated from the examination equipment. The patient was trained to breathe before the scan and held his breath at the end of the inhale. The patient performed a full lung scan with a breathless breath after inhalation, ranging from the tip of the lung to the angle of the ribbed diaphragm. The scanning parameters were as follows: tube voltage 120kV, tube current 225mA, layer thickness 1.5mm, image matrix 512mm×512mm, visual field 360mm×360mm. The reconstruction algorithm was a standard algorithm reconstruction for 1.25mm layer thickness axial image reconstruction. After scanning, the equipment, machine room, and patient passage were disinfected by the hospital sterilization and antivirus team.

### 2.3. Methods of lesion detection in DL-based AI-assisted software

**Fig 1:**
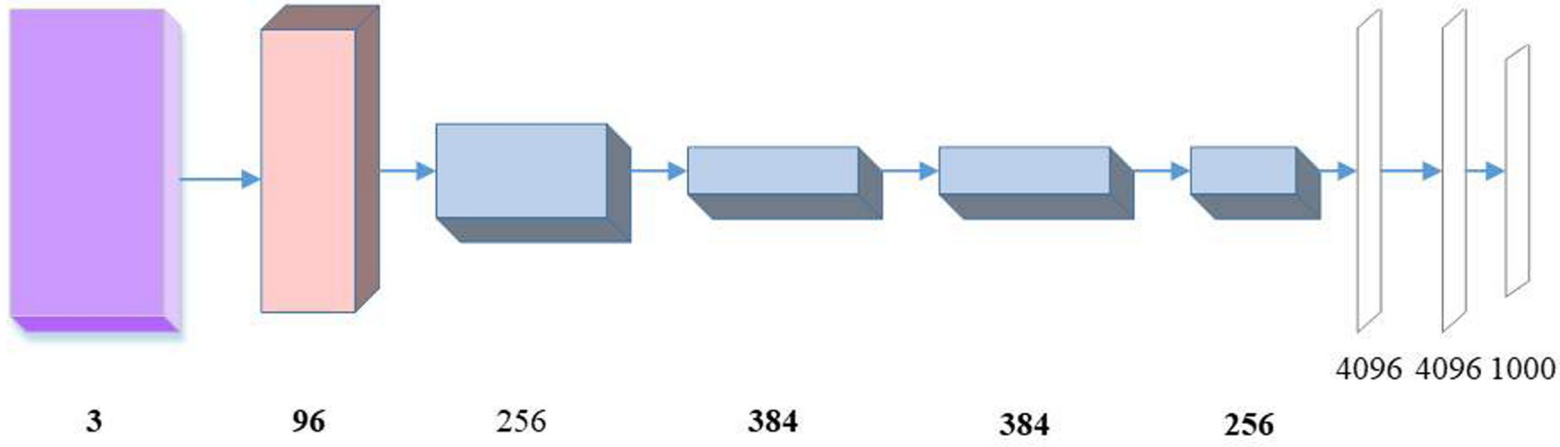
The AlexNet model.

UAI, the pulmonary infectious disease intelligent auxiliary analysis system (uAI-Discover-NCP, Lianying Group), a DL-based AI-assisted diagnostic software was used to detect lesions. The original CT raw data of all pulmonary infectious disease patients were transmitted to the AI software workstation, the software system automatically identified and labeled pneumonia lesions in batches, and presented the calculated data in the form of pictures and charts, including the total volume of lesion, the volume of internal GGO and the volume of the real variable region. The automatically detected target lesions were marked and numbered in boxes on various images, basic information, such as lesion area, volume, length diameter, and CT value were provided, and the corresponding recognized and labeled nodules could be displayed on the sliding image layer at all levels. The local 3D reconstruction map could be rotated in multiple directions to show the anatomical structure of the target lesion and its position relationship with the surrounding tissue. In a small sample pulmonary infectious disease CT data set, each sample contains the gray value and corresponding label information. There are three different sample labels in the pulmonary infectious disease CT data set, which are converted to one hot coding before the model is trained. For image-target detection, the feature extraction methods with different dimensions have been replaced by the DCNN (Deep Convolution Neural Network) that outputs multi-level feature maps. The key to DCNN feature extraction is to mine higher-level semantics and rationally utilize rich semantic information of high-level features and accurate target location information of low-level features. A Single Feature Map is the basic structure of DCNN feature extraction. The image features are extracted through multiple convolution operations, and the receptive fields are gradually increased. Finally, the results are predicted according to the feature map. In the UAI system, the CT images of pulmonary infectious disease are recognized based on the AlexNet model. Feature extraction and classification of CT images of pulmonary infectious disease are conducted through the AlexNet model (Fig 1). The initial learning rate and momentum of the model are set to 0.001 and 0.9 in the training process.

CT images of pulmonary infectious disease are characterized by multifocal disease, low contrast, and unclear. These characteristics make the semantics before and after in network computing more complex and the semantic gap larger. However, in the past, the high and low semantic modes of codec network splicing is relatively simple, and the semantics cannot be integrated well, which easily leads to the loss of pixel features, making the network unable to obtain enough rich feature information for accurate segmentation. A Loop Residual Block (LRB) is designed to reduce the semantic gap. Regarding the LRB, Tang et al. (2022) [16] proposed a semantically aware real-time infrared and visible image fusion network. They discussed image fusion in advanced vision tasks and introduced a new network model capable of automatically combining infrared and visible images to produce richer and semantically aware results. Different from the previous single-branch and single-layer skip connection residual structure, this structure has multiple branches, considering skip connections across multiple layers and in reverse. Skip connections across multiple layers can make the convolutional layer receive semantic features at a longer distance. That is, the feature input of convolutional layers not only includes the input and output of the previous layer but also the input and output of further layers. The reverse skip connection concatenates the feature information and inputs it into the convolutional layer, making the capture of feature information more comprehensive. The whole module acts as a bridge connection layer, connecting the encoder and the decoder. This type of bridge connection not only plays a role in buffering feature semantics and promoting feature fusion of codecs but also obtains richer feature representations. In addition to the LRB, the Alexnet model and Universal Artificial Intelligence (UAI) are important references. The Alexnet model is a classical CNN architecture in DL, which greatly affects image recognition tasks. It introduces innovative designs such as multi-layer convolution, pooling layers, and ReLU activation functions, improving the model’s performance and training speed. In the field of image processing, the use of the Alexnet model for feature extraction and classification has many successful cases. UAI refers to universal AI, aimed at developing AI systems with wide applicability and intelligence [17]. Combining knowledge and technology from different fields achieves more comprehensive and intelligent problem-solving capabilities. The research and development of UAI provide new ideas and methods for improving the accuracy and efficiency of medical image analysis and diagnosis. Meanwhile, UAI has also played an important role in public health management and epidemic control, helping decision-makers develop scientific and effective prevention and control strategies through data analysis and prediction. In summary, the Alexnet model and UAI provide vital references and guidance for research and practice in medical image analysis and epidemic management, and their applications are expected to promote the development and innovation of related fields [18–20].

The Alexnet model consists of 5 convolution layers and 3 fully connected layers, namely, an 8-layer deep network. The output of the last fully connected layer is sent to the Softmax layer, thus producing a distribution covering multi-class labels. Compared with the traditional neural network, the Alexnet model has the following advantages: ① It adopts the ReLu activation function; ② It enhances the data set to suppress overfitting; ③ It employs the Dropout method to suppress overfitting; ④ It uses Local Response Normalization (LRN) to enhance generalization.

The input to the Alexnet model is 227 ×227 × 3 images. This section chooses the three-channel RGB pathological images to meet the model’s dimensional requirements. The readjusted pathological image is sent to the network; first, it reaches the convolution layer; then, after the first layer convolution operation, the results are pooled, normalized, and input into the second layer; likewise, the second layer to the fifth layer performs operations similar to those of the first layer. Afterward, the output of the fifth layer will be sent to the following fully connected layer. The outputs of the sixth and seventh layers are vectors with a length of 1,000. Finally, the final classification results are obtained through the Softmax classifier. The network classification performance is evaluated through Accuracy.

All imaging physicians had received relevant professional training and were familiar with the operation process and related matters of the software. Based on the images shown in the image archiving and communication system, the primary imaging physicians checked the lesion identified by the AI-assisted diagnostic software and recorded the false positive or false negative situation in the software recognition area. Finally, the final review was carried out by two senior experienced diagnostic physicians, a few false positive or false negative images were repaired manually, and only a few required manual edits. All patients underwent clinical treatment for 3 to 5 days before CT reexamination to assess the patient’s disease progression.

### 2.4. Manual detection method

Image measurement and analysis were evaluated by two radiologists with more than 5 years of experience in diagnosis. Consensus could be reached through consultation with a senior radiologist with more than 10 years of experience when opinions differed. Pneumonia performance was evaluated, including GGO, consolidation shadows, mixed density GGO shadow, air bronchus sign, grid shadow, central lobules nodules, and pleural downline samples sign, cystic degeneration, and bronchiectasis, GGO showed increased slight of pulmonary parenchymal density, and the bronchial vascular in the lesion area was still displayed. Consolidation shadows showed increased pulmonary parenchymal density, and the vascular shadow was not visible in the lesion area. The distribution of the lesion was divided into peripheral and central, the peripheral distribution was the lateral 1/3 of the lung, and the central distribution was the medial 2/3 of the lung. Other abnormal pulmonary lesions, such as fibrous changes, nodules, calcification, masses, voids, lymph node lesion, and pleural effusion, were also recorded. The cumulative number of lobes was recorded, and the involvement of a single lobe or multiple lobes was assessed and recorded.

### 2.5. Statistical methods

SPSS 26.0 statistical software was used for data analysis, the measurement data were expressed as mean ± standard deviation ($\bar{x}$±s), t-test was used for the comparison of the two samples, the counting data were expressed as incidence n (%), x^2^ test was used for the comparison, and all of them were statistically significant with P<0.05.

## 3. Results

### 3.1. Basic information on patients

Among the 200 confirmed pulmonary infectious disease patients, 147 were normal patients, 40 were severe patients, and 13 were critical patients. The basic situation of the patients was shown in Table 1, and among the 200 confirmed pulmonary infectious disease patients, 106 were males and 94 were females, aged from 28 to 75 years old, with an average age of (50.34±12.65) years old. The patient’s body temperature at admission was 36.7∼38.5°C. The main clinical manifestations of the patients were shown in Table 2, including 20 cases of expectoration (10.00%), 113 cases of fever (56.50%), 33 cases of chest tightness (16.50%), 140 cases of cough (70.00%), 13 cases of headache (6.50%), 20 cases of pharyngeal pain (10.00%), 27 cases of chills (13.50%), and 7 cases of digestive system symptoms (3.50%).

**Table 1:** The basic information on patients.

Basic information	Value
Gender male [case (%)]	106 (53.00)
Age (years)	50.34±12.65
Body temperature (°C)	37.5±0.8
Heart rate (beat/min)	102±9
Frequency of breath (time/min)	19.3±1.0
Systolic pressure (mm Hg)	150±6
Diastolic pressure (mm Hg)	94±4
Blood oxygen saturation	97.2±0.5

**Table 2:** Main clinical manifestation of patients.

Clinical manifestation	Cases	Proportion
Expectoration	20	10.00%
Fever	113	56.50%
Chest tightness	33	16.50%
Cough	140	70.00%
Headache	13	6.50%
Pharyngeal pain	20	10.00%
Chills	27	13.50%
Digestive system symptoms	7	3.50%

### 3.2. CT manisfestations of patients

**Fig 2:**
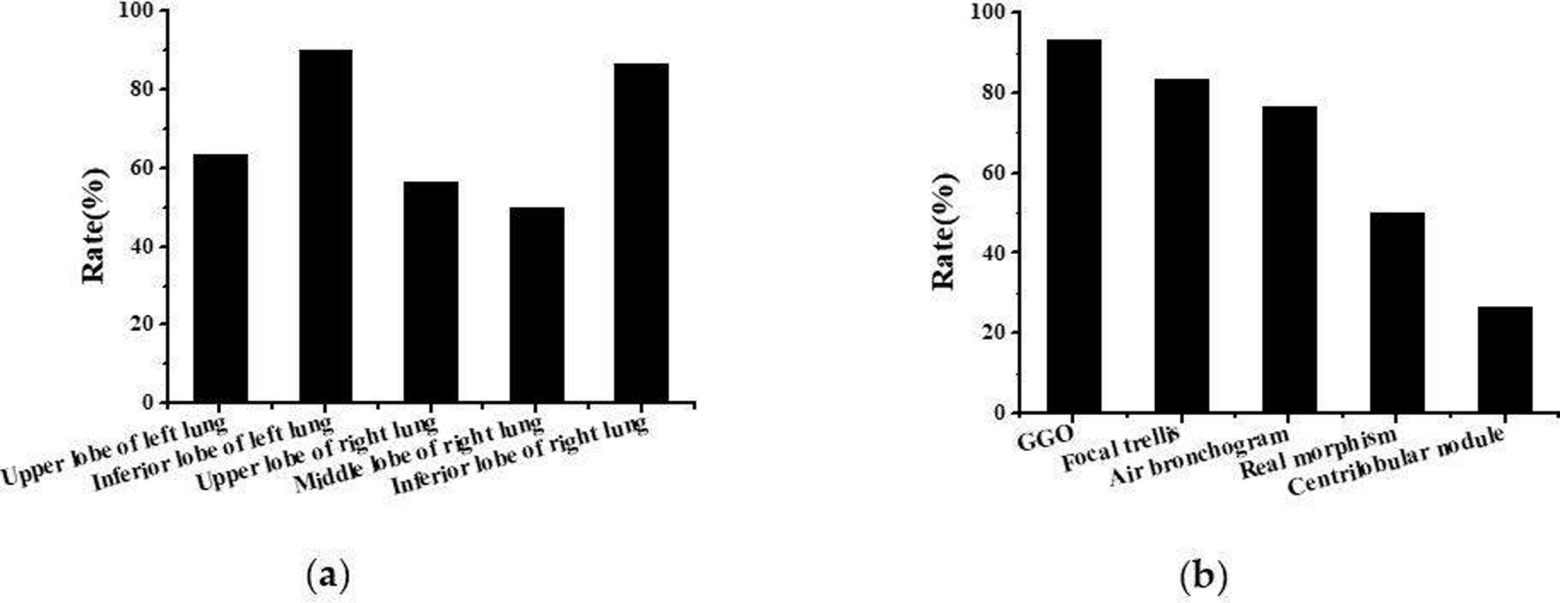
Patients’ CT characterization results: (a) pulmonary lobe involvement; (b) proportion of CT signs.

The CT characterization results of the patient were shown in Fig 2A and 2B. Among the 200 patients diagnosed with pulmonary infectious disease, CT manifestations were mainly multiple lobes involvement, including 127 cases (63.50%) of the left upper lobe, 180 cases (90.00%) of the left lower lobe, 113 cases (56.50%) of the right upper lobe, 100 cases (50.00%) of the right middle lobe, and 173 cases (86.50%) of the right lower lobe. In terms of density, 187 patients (93.50%) developed GGO, 167 patients (83.50%) had focal mesophilic, 153 patients (76.50%) had air bronchus sign, 100 patients (50.00%) had consolidation shadows, and 53 patients (26.50%) had central lobular nodules. No lymphadenopathy and pleural effusion were observed in all patient’s CT examinations.

**Fig 3:**
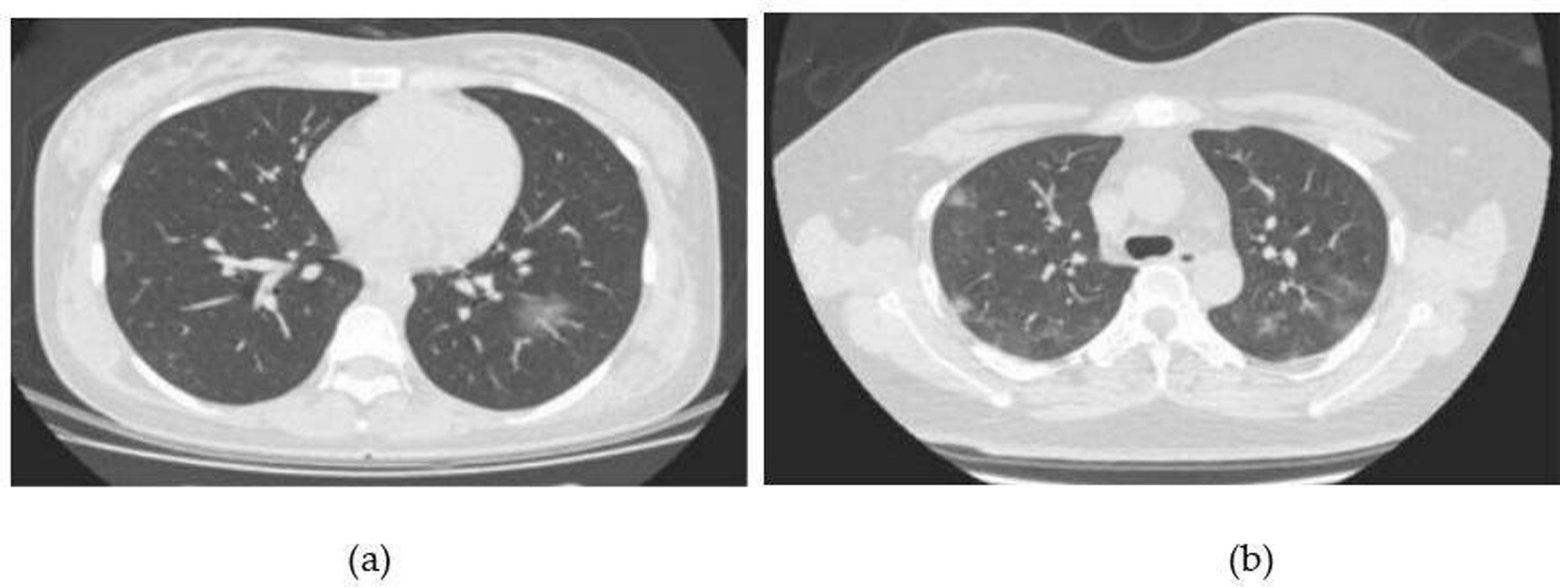
Typical CT images of patients: (a) clinical common patients; (b) the main manifestations of GGO shadows in patients.

Typical CT images of patients were shown in Fig 3. Clinical common type patients CT showed unilateral or double lung multiple lesions, a slice or wedge-shaped GGO, the vessels and bronchi could be seen inside, often accompanied by thickening of the interlobular septum, "paving stones" sign and bronchial inflatable sign, and lung consolidation could be seen inside some lesions. In the typical clinical common case shown in Fig 3A, a CT scan of a patient with pulmonary infectious diseases showed GGO in the peripheral zone of both lower lungs, with a grid shadow seen inside. The CT of clinically severe and critically ill patients showed a wide range of lesions, GGO, solid-shadow, and fiber-strip shadow, with a "paving stone" sign, and bronchial inflatable sign. In Fig 3B, a CT cross-sectional scan of a patient with pulmonary infectious disease in the typical clinical severe case showed the air bronchi sign in the periphery zone GGO of the lower lobe of the right lung.

### 3.3. The evaluation results of patient’s condition by AI software lesion detection method based on the DL model

**Fig 4:**
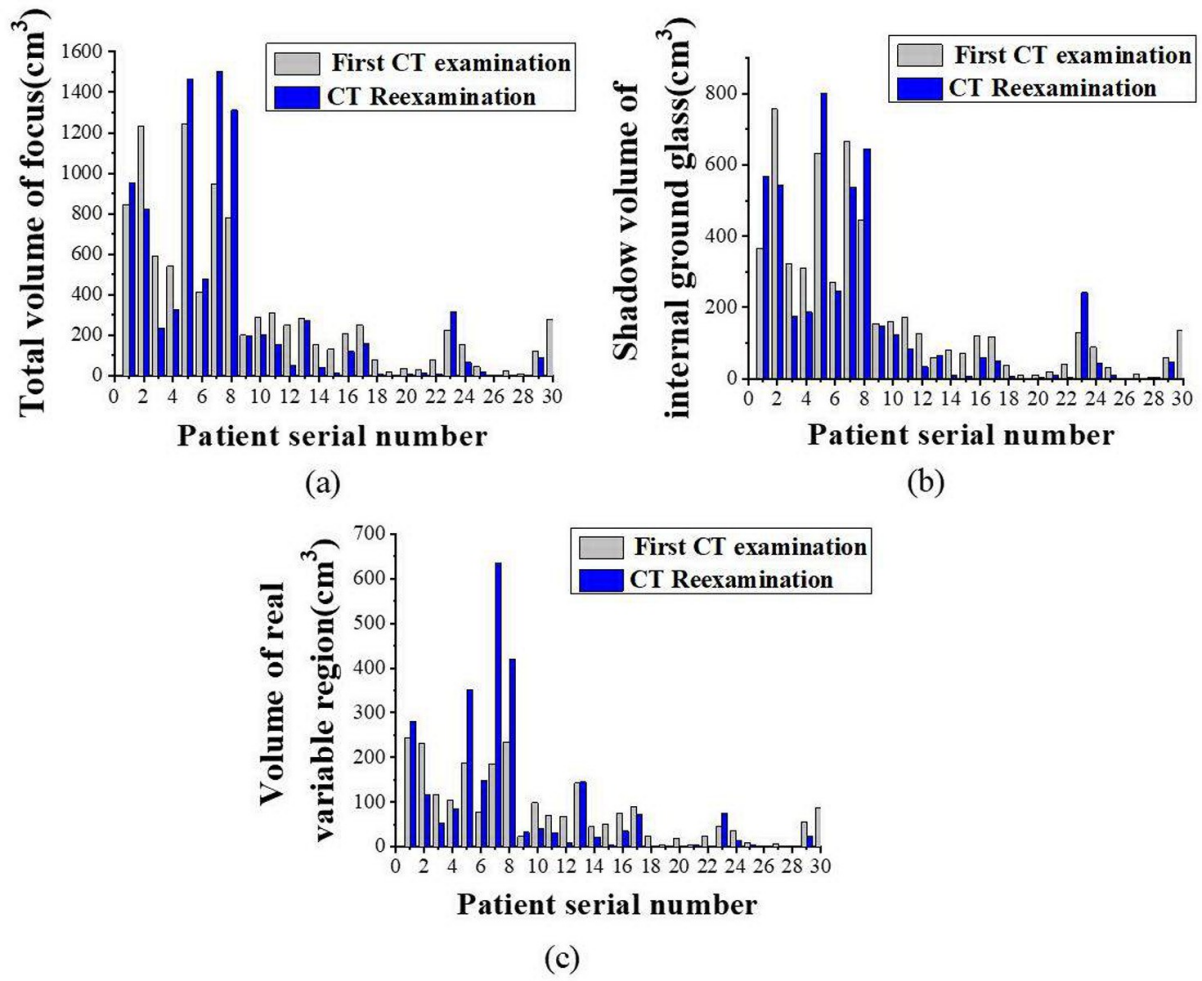
The evaluation results of patient’s condition by DL-based AI-assisted lesion detection method: (a) Total volume of the lesions; (b) Internal GGO volume; (c) the volume of the real variable region. (Note: The time interval between the First CT examination and the CT Reexamination in the figure is 3–5 days).

This paper extracted representative experimental data from 30 clinical patients to analyze the changes in total lesion volume, Internal GGO volume, and the volume of the real variable region. These 30 clinical patients were independently selected from the entire dataset. Their data were used to further validate the effectiveness of the DL-based AI method in clinical practice. The use of these patient data for model training was avoided in this process to ensure the objectivity and independence of the assessment. This selection is based on several criteria, including but not limited to (1) ensuring that the selected patients are clinically representative and reflect the different types and degrees of pulmonary infection lesions. (2) Patients were selected to cover various conditions to evaluate the performance of the DL-based AI method more fully. (3) The CT image data of these 30 selected patients must be complete, clear, and included in the study dataset. The evaluation results of the patient’s condition using the DL-based AI-assisted lesion detection method are indicated in Fig 4A–4C. The AI-assisted diagnostic software could automatically identify and label the pneumonia lesion. The total volume of the patient’s lesion, the shadow volume of internal GGO, and the volume of solid areas were automatically calculated by the software. After 3–5 days of clinical treatment, CT re-examination showed that, among the 200 patients, the total volume of lesion, the volume of internal GGO, and the volume of the real variable region of 21 patients decreased accordingly. In 6 patients, the total volume of lesions, the volume of internal GGO, or the volume of the real variable region increased accordingly; while in 3 patients, the total volume of lesions, the volume of internal GGO, or the volume of the real variable region did not change significantly.

**Fig 5:**
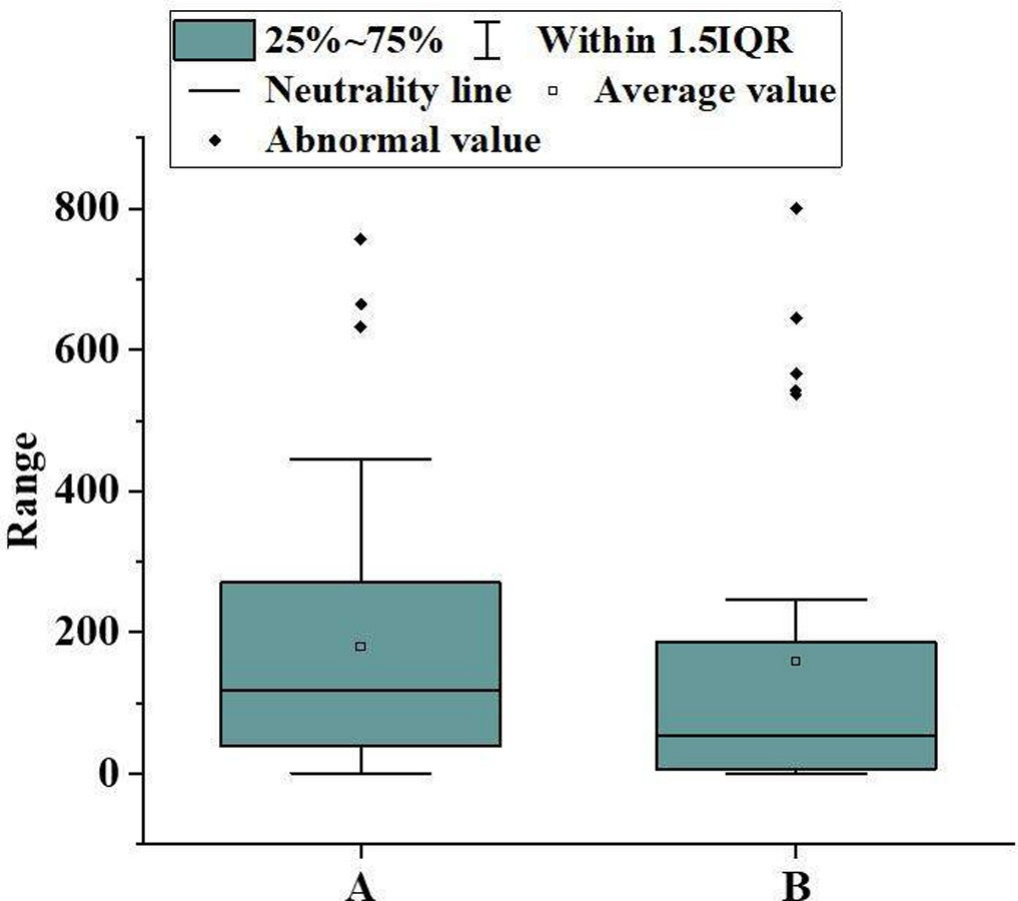
Box plot of the total volume change of lesions in two CT examinations.

Fig 5 uses a box plot to compare the changes in the total volume of the lesions of 30 representative patients during the first CT examination and follow-up after 3–5 days of clinical treatment. The horizontal axis in the figure represents the first and follow-up CT examinations, while the vertical axis represents the total volume of the lesion. The box refers to the quartile spacing, the distribution range of samples between 25% and 75%. The thin lines inside the box represent the median, the upper and lower two lines represent the upper and lower quartile values, and the dots indicate outliers. Fig 5 denotes that the median of the total lesion volume of the patient at the first examination was about 15cm^3^. After treatment, the median of the total lesion volume decreased to about 10cm^3^ during follow-up, and the range of the middle 50% sample was also narrowed. During the follow-up examination, some abnormal values of increased lesion volume were observed. Overall, between the two CT examinations, the total volume of the lesion showed a decreasing trend, indicating that the majority of patients have improved their condition. This confirms that DL-based AI-assisted methods can effectively assess changes in patients’ condition. However, in some cases, the increase in lesion volume indicates the progression of the disease, and close monitoring is still needed in clinical practice.

### 3.4. Comparison of two methods for lesion detection

**Fig 6:**
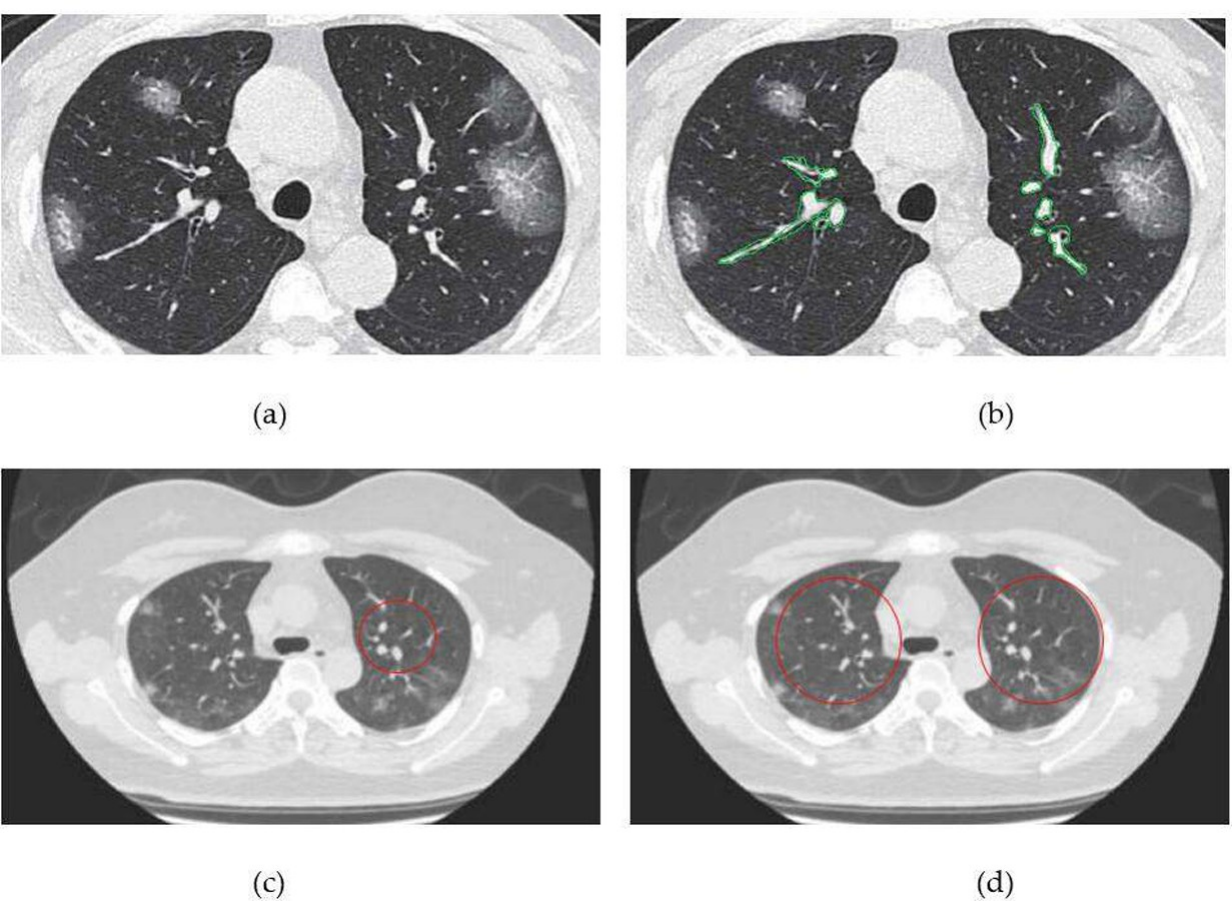
Comparison of lesion marker ranges between the two methods: (a) The original image; (b) An image of the lesion manually drawn by experts; (c) DL-based AI-assisted lesion detection method; (d) Manual testing method.

The comparison results of the lesion marker range between the two methods are shown in Fig 6. The gold standard for diagnosing pneumonia is invasive lung lesions, which are aggressive lesions in the lungs, as it shows in Fig 6A. An image of the lesions manually sketched by experts is depicted in Fig 6B. It could be seen that the lesion marker range of the DL-based AI-assisted lesion detection method (Fig 6C) is more consistent than that of the manual detection method (Fig 6D).

Fig 6 compares the lesion marker ranges of two methods. It can be seen that the DL-based AI-assisted has a more consistent lesion range. For the right side of Fig 6, the range of the AI method is relatively small. Considering that its detection ability is superior to the manual methods mentioned in this paper, this range is more likely to represent real lesions. On the left side, the AI method does not detect a certain area, which may be an omission, reflecting the presence of false negatives. Manual methods often detect certain areas, which may be false positives and reflect the presence of false positives. Regarding the doubts about the results in Fig 6, after discussion, it is believed that for the right side, the AI method has a smaller range that is more accurate and reflects a stronger detection ability. For the left side, there are omissions in the AI method and false positives in the manual method, which means there are certain false negatives and false positives. Here, 200 patients were identified, with a total of 2533 lesions identified. The number of non-lesion areas is approximately 37000. The AI method still performs better than the manual method, but there is room for improvement, such as model optimization to reduce false negative and false positive results. This fully indicates that in lesion detection, the effectiveness of the testing method cannot be solely based on the detection rate but also needs to pay attention to false positives and negatives to evaluate the method’s advantages and disadvantages objectively.

**Fig 7:**
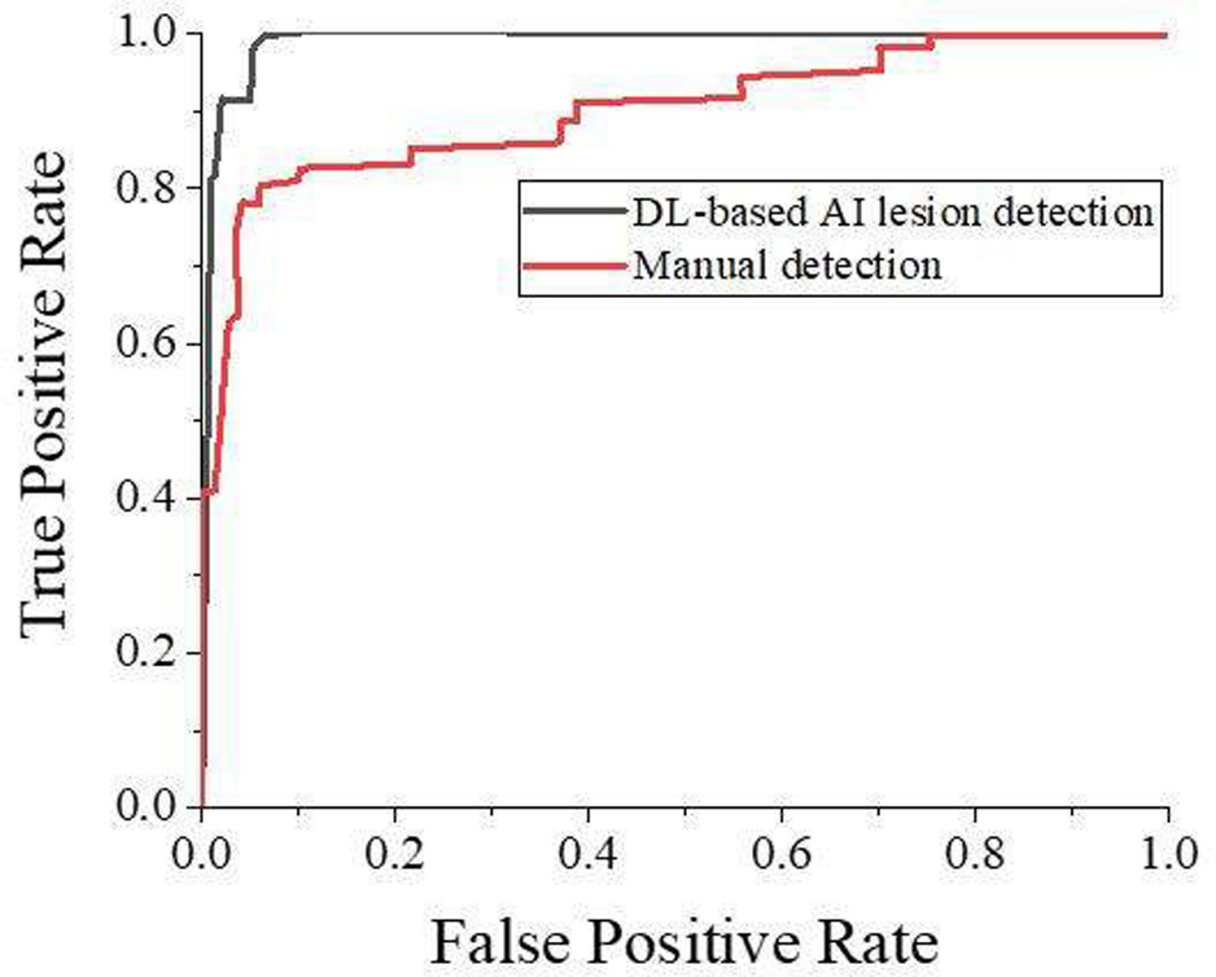
The comparison results of the ROC curves of the two methods.

There were 2533 lesions in 200 patients. Table 3 displays lesion detection under the two methods. In this paper, 2533 represents the total number of real pulmonary infections. This includes True Positive lesions successfully detected by AI, and False Negative lesions missed by AI. This number is based on the "gold standard" of clinical image evaluation and represents the actual presence of pulmonary infectious lesions. In Table 3, "Misdetection of lesions" refers to AI’s misidentification of normal tissues or structures due to AI mistakenly identifying non-lesion areas as lesions. False positives are not part of the actual lesion, but rather errors in AI detection. This number will not be included in the total number of real lesions. The "gold standard" results from an independent evaluation of clinical images by a professional physician and is considered an accurate and objective standard. Manual detection is the result of the evaluation of the same set of images by a doctor. In medical research, the "gold standard" is often considered a reference standard because it is based on the consensus and experience of professional physicians and can provide a more accurate diagnosis. "Missed lesions" refer to real lesions that cannot be detected by AI, i.e. missed detections by AI. However, in the "Misdetection of lesions" column, the number of lesions incorrectly detected by the AI is described. Here, the "Misdetection of lesions" is not really lesions, but rather that the AI is mislabeling normal tissue or structure as lesions, so this is part of the misdetection. The total number of lesions is the number of true lesions (the sum of those detected and missed by AI) and the number of false lesions detected by mistake. Therefore, to be precise, 2533 "real lesions" cover 2527 real lesions detected by AI, as well as 4 real lesions missed by AI and 2 non-real lesions incorrectly detected by AI. Under the DL-based AI-assisted method, 2527 lesions were detected, and 4 lesions were missed, without errors. While under manual detection, 2414 lesions were detected, among which 4 lesions were miss-detected, and 114 lesions were missed. Meanwhile, the lesion detection rate, the misdetection rate, and the missed diagnosis rate of the DL-based AI-assisted lesion detection method were 99.76%, 0.08%, and 0.16%, respectively. The lesion detection rate, the misdetection rate, and the missed diagnosis rate of manual detection were 95.30%, 0.20%, and 4.50%, respectively. Hence, the detection rate of the DL-based AI lesion detection method was significantly higher than that of manual detection, while the rate of misdetection and missed diagnosis was significantly lower than that of the manual detection method, and the difference was statistically significant (P<0.05). Fed with abundant amounts of data, the DL model can well-assist doctors in pulmonary infectious disease CT diagnosis by improving the CT recognition accuracy and stability. The comparison results of the ROC curves of the two methods are portrayed in Fig 7.

**Table 3:** Lesion detection by the two methods.

	DL-based AI lesion detection	Manual detection	P
Lesion detected	2527	2414	
Detection rate	99.76%	95.30%	0.035
Missed lesions	4	114	
Missed diagnosis rate	0.16%	4.50%	0.022
Misdetection of lesions	2	5	
Misdetection rate	0.08%	0.20%	0.013

Note: P<0.05 indicates statistical significance, Total lesions N = 2533

The data in Table 3 is based on lesion detection and evaluation of lung infection disease images collected by the research institute. The DL-based AI-assisted and manual detection methods were applied to the same data set, and their lesion-detection effects were compared. Specifically, the paper used images from 200 patients as an evaluation data set, which included 2533 lesions. Firstly, the research team employed data from 200 patients with pulmonary infectious diseases as the overall data set. These data are divided into training and testing data sets for model training and performance evaluation. Training data set: For this data set, the research team selected 80% of the images, totaling 160 images, for training the DL model. During the training process, image preprocessing and enhancement operations are performed to improve the generalization ability and accuracy of the model. These operations involve image graying, equalization, filtering, etc. Test dataset: The remaining 20% of the images, consisting of 40 images, were used as the test dataset for the model. The p-values of detection rate, missed diagnosis rate, and misdiagnosis rate in Table 3 were obtained through statistical methods. Regarding statistical methods, the detection rate adopts the chi-square test of independent sample rate, while the missed diagnosis rate and misdiagnosis rate use the Fisher exact test of independent sample rate. The chi-square test is suitable for comparisons with larger total sample sizes, while the Fisher exact test is suitable for comparisons with smaller ones.

There are also differences between the two types of false detections in Table 3. Missed diagnosis refers to the failure to detect the true and existing lesions that should have been detected and belong to false negative results. Misdiagnosis determines the absence of lesions as the presence of lesions, which is a false positive result. The difference between the two is that the former appears in true positive samples, while the latter appears in true negative samples. During the testing process, the research team utilized the DL-based AI-assisted and manual detection methods to detect lesions in these images and compared the performance of the two methods. In addition, by dividing the data set into multiple subsets, cross-validation can be used to train and validate the model multiple times, thereby evaluating the model’s performance more comprehensively. In summary, appropriate statistical methods can significantly test the advantages and disadvantages of two lesion detection methods. Table 3 verifies that the DL-based AI-assisted diagnosis can improve the detection rate, and reduce missed diagnosis rate and misdiagnosis rate. Understanding the differences in types of false positives can also help analyze the sources of detection errors for method improvement. The data results of the training set and test set are listed in Table 4. In terms of the training set, DL-based AI lesion detection shows a high detection rate, reaching 98.8%. In comparison, the detection rate of manual detection is 94.6%. This means that in the training set, the DL-based AI method is more effective than manual detection in detecting pulmonary infection lesions in patients. Through the analysis of 2022 training set images, the DL method misses only 1 image, achieving an accuracy of up to 98.60%. The sensitivity and specificity are 95.9% and 96.5%, respectively. This indicates that the DL-based AI performs well on the training set, accurately identifies pulmonary infection lesions, and has higher efficiency than manual detection. Regarding the test set, DL-based AI lesion detection also presents excellent performance, with a detection rate of 99.76%, while manual detection has a detection rate of 95.30%. By analyzing 504 images in the test set, the DL method misses only 2 images, and the accuracy reaches 98.70%. The sensitivity and specificity of the test set are 93.10% and 91.80%. Although the DL method has a slight decline in the test set compared with the training set, it still shows a higher accuracy and detection rate and has better performance than the manual detection method. The significance level of the P-value is 0.032, indicating that the performance difference between the two methods on the test set is remarkable. In summary, DL-based AI lesion detection exhibits a higher detection rate and accuracy in both training and test sets and has obvious advantages over manual detection methods. This provides a more effective and reliable auxiliary method for diagnosing pulmonary infection.

**Table 4:** The data results of training and testing sets for pulmonary infection diseases.

	Patients	DL-based AI lesion detection (detection rate)	Manual detection (detection rate)	Detected lesion image	Missing images	AUC	Accuracy	Sensitivity	Specificity	P-value
Training set	160	98.8%	94.6%	2022	1	98.60%	95.9%	96.5%	95.7%	0.018
Test set	40	99.76%	95.30%	504	2	98.70%	93.10%	91.80%	94.60%	0.032

Fig 7 demonstrates the comparison results of two detection methods for ROC curves. The ROC curve can comprehensively reflect the true positive rate and false positive rate under different detection thresholds, and evaluate the effectiveness of detection methods. Generally speaking, the larger the area value below the ROC curve, the better the performance of the detection method. Fig 7 shows that the ROC curve of the DL-based AI-assisted method is better than that of the manual detection method, indicating that the former has better detection efficiency.

Moreover, the false positive rate is an important indicator for evaluating the specificity of detection methods. It represents the proportion of samples without lesions that are misdiagnosed as lesions. Ideally, the false positive rate should be close to 0. Table 3 details that the DL-based method’s false positive rate (i.e., misdiagnosis rate) is 0.08%, lower than the 0.20% of manual detection, illustrating that the former has better specificity.

In short, using ROC analysis and false positive rates can evaluate the effectiveness of detection methods. The results of this paper indicate that DL-based AI-assisted detection methods can offer higher sensitivity and specificity than manual detection. This provides support for the clinical application of the AI system. However, the detection efficiency can still be improved, and further research can collect more sample data and optimize the model to improve ROC further and reduce false positive rates.

## 4. Discussion

DL techniques can effectively detect, recognize, and classify images, so the introduction of DL techniques in the field of imaging may facilitate radiologists with medical detection and diagnosis [21, 22]. Pulmonary nodule detection using an AI algorithm is an important part of the AI medical field [23]. The results indicated that the two detection methods had the same range of lesion markers. However, the detection rate of the AI detection method based on DL was significantly higher than that of the manual detection method (P<0.05), while the false detection rate and missed diagnosis rate were remarkably lower than that of the manual detection method (P<0.05) [24].

To sum up, the false detection rate and missed diagnosis rate of DL-based AI-assisted diagnostic software are higher than that of imaging physicians’ manual detection, and the detection ability still needs to be improved. Overall, however, AI-assisted diagnostic CT can well-detect lesions in pulmonary infectious disease patients, and can effectively identify the lesions, provide relevant data information, such as the total volume of the lesion, the internal GGO, and the volume of solid change, and show the variation of lesion range and internal density. It provides objective imaging support for pulmonary infectious disease diagnosis and public health management but also improves the efficiency of imaging physicians.

Pulmonary infectious disease patients are studied to explore the application of AI in diagnosing coronavirus pneumonia and public health management. The results show that the AI-assisted diagnosis CT can effectively identify pulmonary infectious disease lesions, provide information about the total volume of lesions, the internal GGO, and the volume of the real variable region, and show the variation of lesion range and internal density. For the diagnosis of pulmonary infectious disease and the management of public health, the reference basis is provided by the experiment, which has important theoretical significance and application value. There are still some shortcomings in the research process, due to the constraints of conditions, the sample data collection is less, resulting in a certain degree of deviation in the results. Therefore, the data capacity will be further increased in the later research process, so that the results obtained are more valuable for reference.

## Supporting information

S1 Data (XLSX)
